# A GIS-based interactive map enabling data-driven decision-making in Nigeria's food supply chain

**DOI:** 10.1016/j.mex.2023.102047

**Published:** 2023-02-01

**Authors:** Divinefavour Odion, Kanaha Shoji, Roberta Evangelista, Joaquin Gajardo, Thomas Motmans, Thijs Defraeye, Daniel Onwude

**Affiliations:** a Constructor University Bremen, Campus Ring 1, 28759 Bremen, Germany; b Empa, Swiss Federal Laboratories for Materials Science and Technology, Laboratory for Biomimetic Membranes and Textiles, Lerchenfeldstrasse 5, CH-9014 St. Gallen, Switzerland; c BASE, Basel Agency for Sustainable Energy, Elisabethenstrasse 22, Basel CH-4051, Switzerland

**Keywords:** Data visualization, Google Earth Engine, Smallholders, Fresh produce, Postharvest supply chain, Cooling solution, Visualizing the potential for sustainable cooling solutions across Nigeria via an interactive web-GIS tool

## Abstract

The accessibility of open-source data on fresh food supply chains provides key stakeholders from the public and private sectors with insights for better decision-making to drive food loss reduction. Nigeria has a fair amount of open-source agricultural and climate-related data. However, most of these datasets are not readily accessible. This paper presents a detailed method used to develop an interactive web Geographic Information System (GIS) tool that collates and visualizes available open-source datasets on Nigeria's Agricultural Sector with particular focus on the fresh produce supply chains. The following steps were used to create such an interactive map.

• Open-source data were acquired in various forms, including tabular, vector, and rasters, processed and uploaded as layers on the interactive web map.
• Most of the data needed some processing on open-source geographic information system applications and web-based computing platforms to transform them into sources of actionable insights
• These final processed layers were then uploaded to a consolidated interactive web map built on the Google Earth Engine platform.

The gathered open-source data includes crop production data, market prices, weather, road network, market locations, mobile coverage, water access, water scarcity, and food insecurity. The method described here also allows the reproduction of such maps for other countries.

## Graphical abstract

Graphical overview of the method presented.

**Figure:**
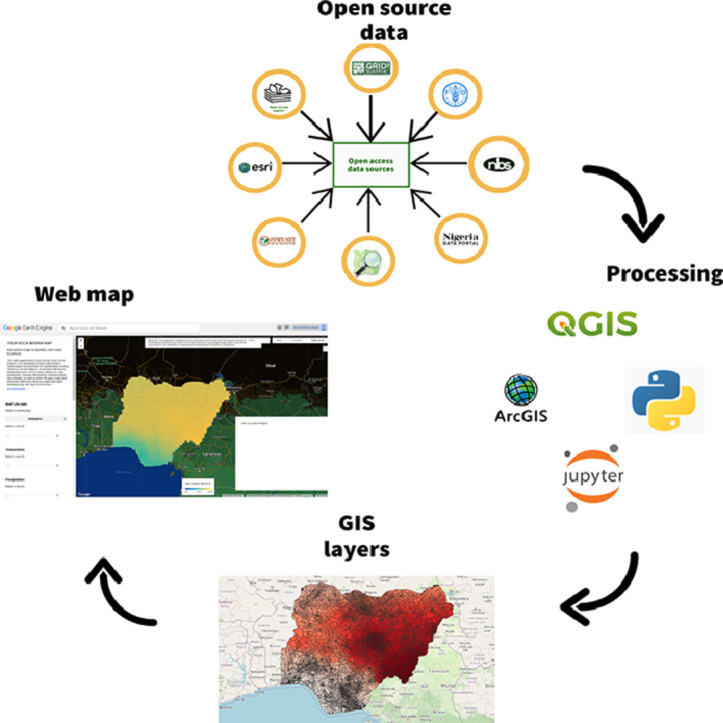

Specifications table

**Table:** 

Subject area	Agricultural and Biological Sciences

More specific subject area:	Fresh food supply chain
Method Name:	Visualizing the potential for sustainable cooling solutions across Nigeria *via* an interactive web-GIS tool
Name and reference of the original method:	Our method was adapted from an early tool created for India: Increasing accessibility and usability of open-source data through a web map for better decision-making in India's cold chain of fresh produce: K. Shoji, R. Evangelista, J. Gajardo, T. Motmans, T. Defraeye, Increasing accessibility and usability of open-source data through a web map for better decision-making in India's cold chain of fresh produce, (2022). https://doi.org/10.5281/ZENODO.6448695.
Resource availability:	Google Earth Engine (GEE), QGIS (version 3.24 Tisler), ArcGIS Pro (version 2.9), Jupyter notebook, Google Colab, R (version 4.1.0), Python (version 3.8.3), etc.

## Introduction

The agricultural sector represents approximately 22% of Nigeria's total Gross Domestic Product and about two-thirds of the labor force [1], yet the country experiences more than 40% loss of fresh produce post-harvest [2]. In Nigeria, the majority of fresh produce farms are located in the Northern region, and as such, fresh produce is transported through road networks to the markets in the southern region where they are consumed. Post-harvest loss occurs in the fresh food supply chain mainly due to inadequate cold storage and refrigerated transportation facilities when these products are being transported from the farms in the North to markets in the southern region. Access to actionable data is a key component needed for implementing digital solutions that can help drive the reduction of these post-harvest losses. Open-access agricultural data, when utilized properly, could empower stakeholders to make their own decisions based on insights and stories embedded in the data. These stakeholders include smallholder farmers, cooling service providers, NGOs, financial institutions, policymakers, and government bodies. However, there is a disconnect between the rising tide of information and the ability of most farmers and other stakeholders in the value chain to use such tools to positively influence the agricultural sector. One way to address this challenge is to make these data broadly accessible to the public *via* an interactive web-GIS tool. Availability of resources such as the interactive web map discussed in this paper will go a long way in mitigating this post-harvest loss.

## Method details

The web map comprises several GIS layers providing relevant information with actionable insights, the synergy of having all these data available is huge for decision-makers. These layers include elevation, electrical grid, population, air temperature, solar radiation, precipitation, road network, market locations, mobile coverage, land cover, agro-ecological zones, food insecurity, blue water scarcity, water access, and crop production. This paper discusses the process of building an openly accessible Google Earth Engine (GEE) application. We highlight each layer on the web map and its sources and go into detail on the processing steps undergone in transforming the raw datasets into layers ready to convey information to stakeholders.

## Data preprocessing steps

The open-source data we collected had various spatial coverage, temporal timespan, and data formats. The processing steps briefly discussed below were necessary to take these differences into account in order to produce layers to be displayed on the web map layer.

### Global data preprocessing

**Fig. 1:**
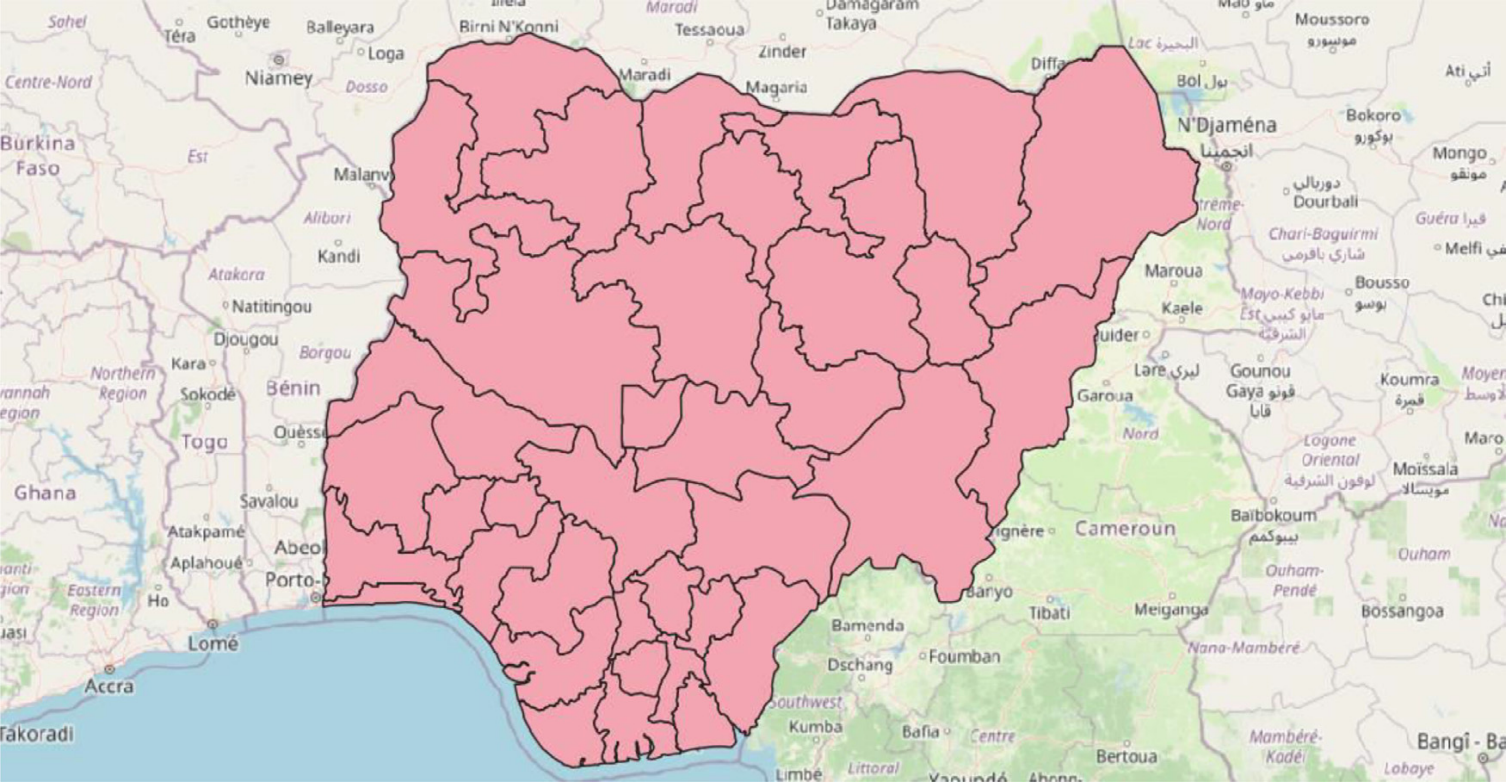
Shapefile of Nigeria map showing state administrative boundaries (from the year 2020). The shapefile was sourced from Ref. [3].

Some open-source data collected consist of global data and therefore needed to be clipped to the extent of Nigeria. The datasets that fall under this category include; climate data (Section 3.3.1, downloaded from https://cds.climate.copernicus.eu/cdsapp#!/dataset/reanalysis-era5-single-levels-monthly-means?tab=form), elevation data (Section 3.3.2, from https://developers.google.com/earth-engine/datasets/catalog/JAXA_ALOS_AW3D30_V3_2), water scarcity (Section 3.3.3, sourced from https://waterfootprint.org/en/resources/waterstat/water-scarcity-statistics/), mobile broadband coverage data (Section 3.3.5, see https://data.apps.fao.org/map/catalog/srv/eng/catalog.search;jsessionid=E9AC46EFCFE2DF24A8F651C17393134A?node=srv#/metadata/b96df310-78f7-4124-83c2-2ee79fb9b554), predicted electricity network lines (Section 3.3.6, see https://zenodo.org/record/3628142#.Y0WA5XZBzb0), agro-ecological zones (Section 3.3.10, downloaded from, https://gaez.fao.org/pages/data-viewer), and land cover (Section 3.3.11, downloaded from https://www.arcgis.com/apps/instant/media/index.html?appid=fc92d38533d440078f17678ebc20e8e2). These layers were clipped to Nigeria using a shapefile with state boundaries from 2020 [3] (Fig. 1). Note that we used these boundaries as they were the latest ones we could find. The elevation data were sourced from the Google Earth Engine data catalog [4] and were directly clipped for Nigeria in the GEE's code editor. The predicted electricity network lines and mobile broadband coverage data were processed in QGIS (version 3.18.2) [5]. The land cover, agro-ecological zones, and blue water scarcity data were processed in QGIS (version 3.24 Tisler) [6] and ArcGIS Pro (version 2.9) [7]. The climate data were processed in R (version 4.1.0) [8].

### Tabular data preprocessing

Some open-source data collected consists of tabular data containing metrics relative to a given state in each row. This is the case for crop production data sourced from the National Agricultural Sample Survey (NASS) of 2010 (see Section 3.3.9), water access levels data sourced from Ref. [9] (see Section 3.3.4), and market price data sourced from the National Bureau of Statistics (NBS) (see Section 3.3.14). The crop production, market prices, and water access levels data were merged with the administrative layer at the state level (as of 2020) [3].

The merging was performed in Python (version 3.8.3) using the ‘FuzzyWuzzy’ (version 0.18.0) library [10] to match state names that might be spelled differently in the different data sources.

### GIS layers

This section describes the layers that are shown on the interactive map and how, when utilized properly can significantly improve the situation of the fresh-food supply chain in Nigeria. The climate layers including information on temperature, precipitation and solar radiance are useful for smallholders in selecting regions in Nigeria with suitable climatic conditions for the crops they produce wherein a lack of this climate information provided in such a synergized manner would see that food waste continues as smallholders make uneducated decisions in this regard. Farmers who grow special vegetables such as carrots which are best grown at relatively higher altitudes can utilize the elevation layer in mapping out good regions to farm. The relevance of the water scarcity layer is highlighted in fresh-food production, this layer will enable effective mapping of regions likely to experience drought during different times in the year. Smallholders empowered with such information will know locations with sufficient ground water to farm. Access to the mobile coverage layer equips cooling providers who monitor and control the temperatures in their hubs remotely with insights that will enable them scale their services efficiently by installing cold rooms in locations with good Network coverage. Furthermore, the information provided in the food insecurity layer is most relevant to the likes of policy makers, NGOs, and Government officials for making good policies that affect the entire fresh food supply-chain. Implementing informed policies can reduce the risk of famine in otherwise stressed areas in the country. The Roads layer on the GIS map comprises of both major roads and dirt roads situated in the country, offering access route information to stakeholders to encourage speedier transportation of farm commodities from the Northern regions to the markets where they are distributed. Mapping out the electricity grid in Nigeria can be important for providers of non-solarized cooling solutions in the country. From the predicted electricity network layer stakeholders can deduce regions with adequate supply of energy required to run their cooling storage facility without leading to spoilage of commodities caused by power outages. The market price chart on the map is useful for analysis of the trends in the market price of some fresh products such as tomato. Knowing how the prices have varied in recent times can be useful for various kinds of stakeholders. For example, a farmer can decide on regions to sell his commodity to based on price information, a policy maker can choose to implement favourable policies for individuals living in areas where the price of certain commodities has experienced a surge etc. The synergy of all these information in one consolidated repository with adequate visualisations is a quick-win for the stakeholders of the fresh-food supply chain of Nigeria. Users can access the map including all layers listed in the sections below *via* the link https://yourvcca-maps.users.earthengine.app/view/yourvcca-map-nigeria .

#### Climate layers

This group of layers was downloaded from the ERA5 database [11]. The names of the extracted parameters are Total precipitation, 2 m temperature, and Total sky direct solar radiation at the surface for total precipitation, temperature, and solar radiance, respectively. These data represent temperature, solar radiance, and total precipitation for each month in 2021. Details about the individual climate layers are discussed below.

##### Precipitation layer

This layer shows the total monthly averaged precipitation expressed as a combination of large-scale and convective rainfall and snowfall rates for the year 2021.

##### Temperature layer

This layer displays the monthly averaged air temperature data for the year 2021 measured at 2 m above the surface of land, sea, or inland waters.

##### Solar radiance layer

This layer displays the monthly averaged direct and diffuse solar radiation accumulated for a single day per month as measured on the earth's surface in W m^−2^.

#### Elevation layer

This layer displays the distance above sea level for the entire country. The data used in creating the elevation layer was sourced from the Japan Aerospace Exploration Agency (JAXA) Earth Observation Research Center [12].

#### Blue water scarcity layer

The water scarcity layer expresses the levels of blue water (fresh surface water and groundwater) scarcity in Nigeria on a monthly basis at a spatial resolution of 30 arc minutes (approximately 60 km). The datasets were sourced from Ref. [13].

#### Water access layer

This layer displays household water and sanitation access levels across the country's 36 states, including the Federal Capital Territory. For this project, we selected four water indicators from the research effort [9], which include “Improved water”, “Basic water”, “Improved source < 30 min trip”, and “Piped water on premises”. Note that for the indicators “Basic water” and “Improved source < 30 min trip” there was no data for Zamfara state.

#### Mobile coverage layer

This layer represents the global mobile broadband coverage for Nigeria at a spatial resolution of 1 km. The datasets were sourced from Ref. [14].

#### Predicted electricity network lines layer

This layer displays Nigeria's predicted electricity network lines as computed by Arderne et al. [15]. The layer was derived *via* a combination of OpenStreetMap data and satellite imagery of Night time lights (See Fig. 4 ).

**Fig. 2:**
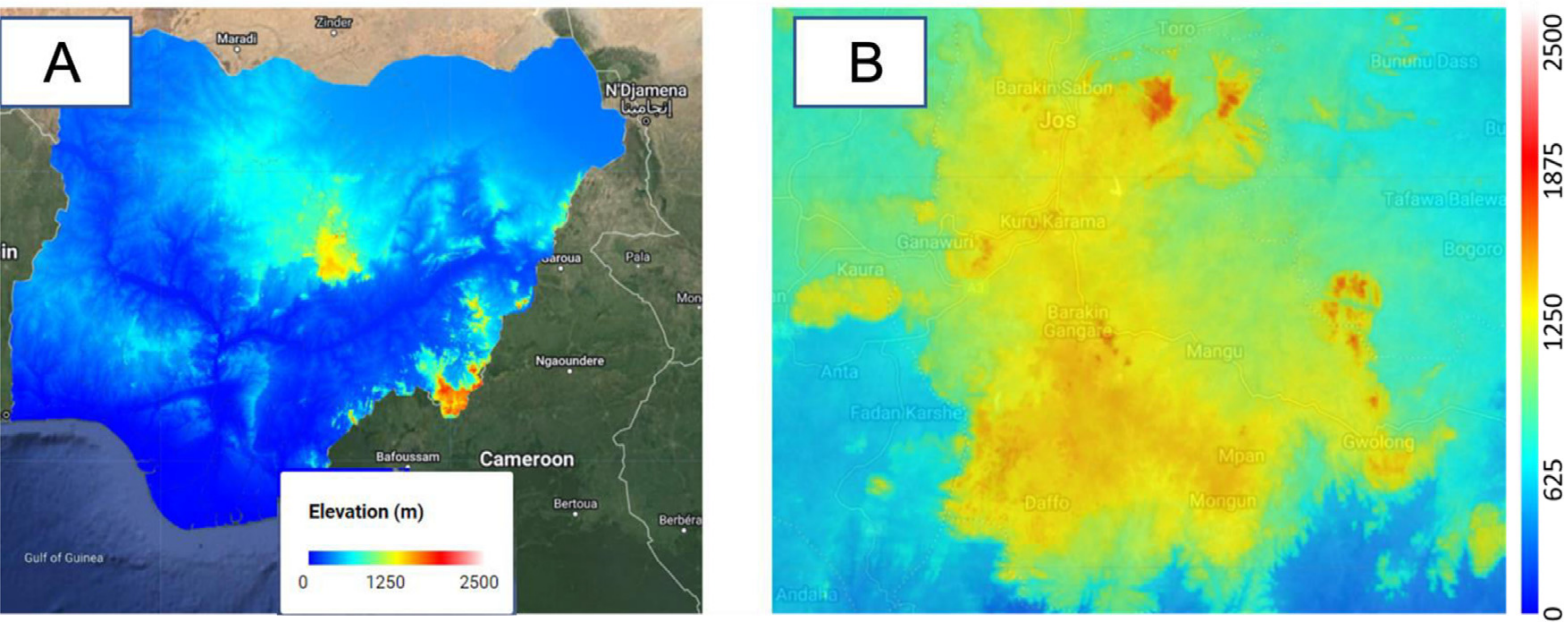
Elevation layer (A: Nigeria, B: Plateau state (Terrain background with 80% opacity)). The global elevation dataset was imported into GEE from Ref. [12].

**Fig. 3:**
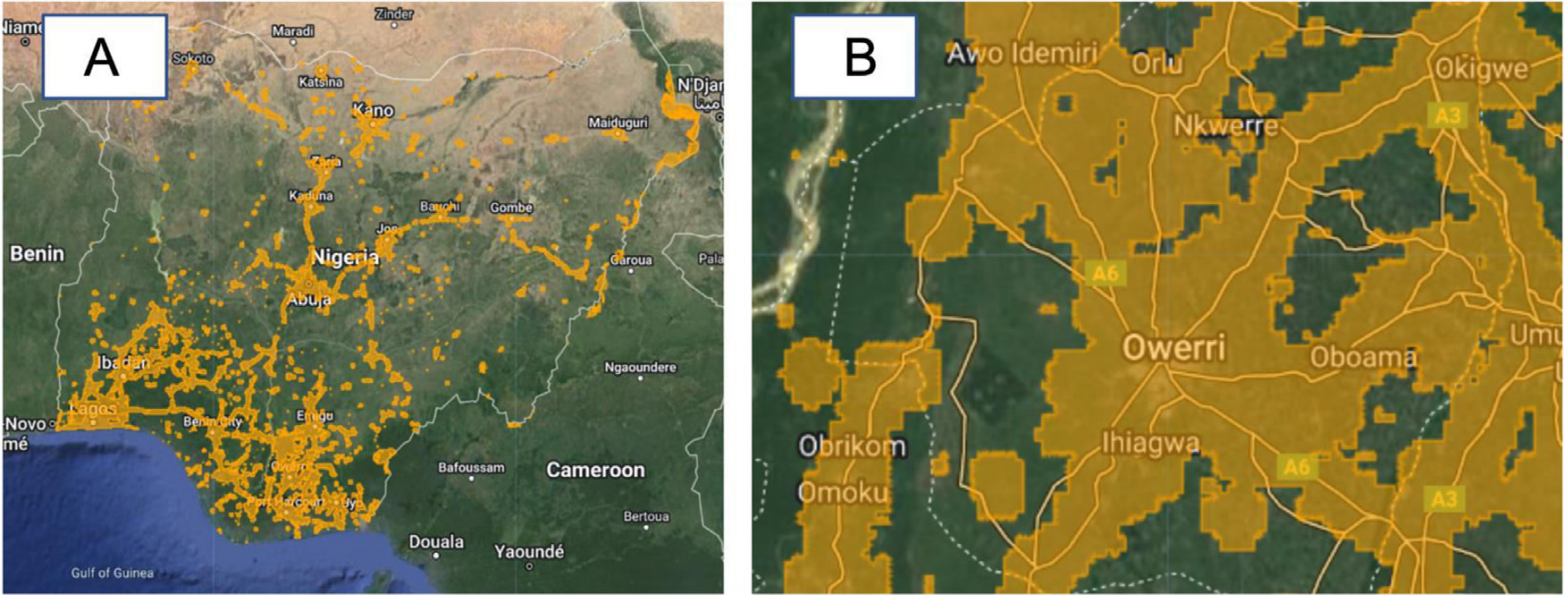
Mobile coverage layer (A: Nigeria, B: Imo state (Terrain background with 80% opacity)). The medium-dark yellow polygons represent the areas with mobile coverage as computed in Ref. [14].

**Fig. 4:**
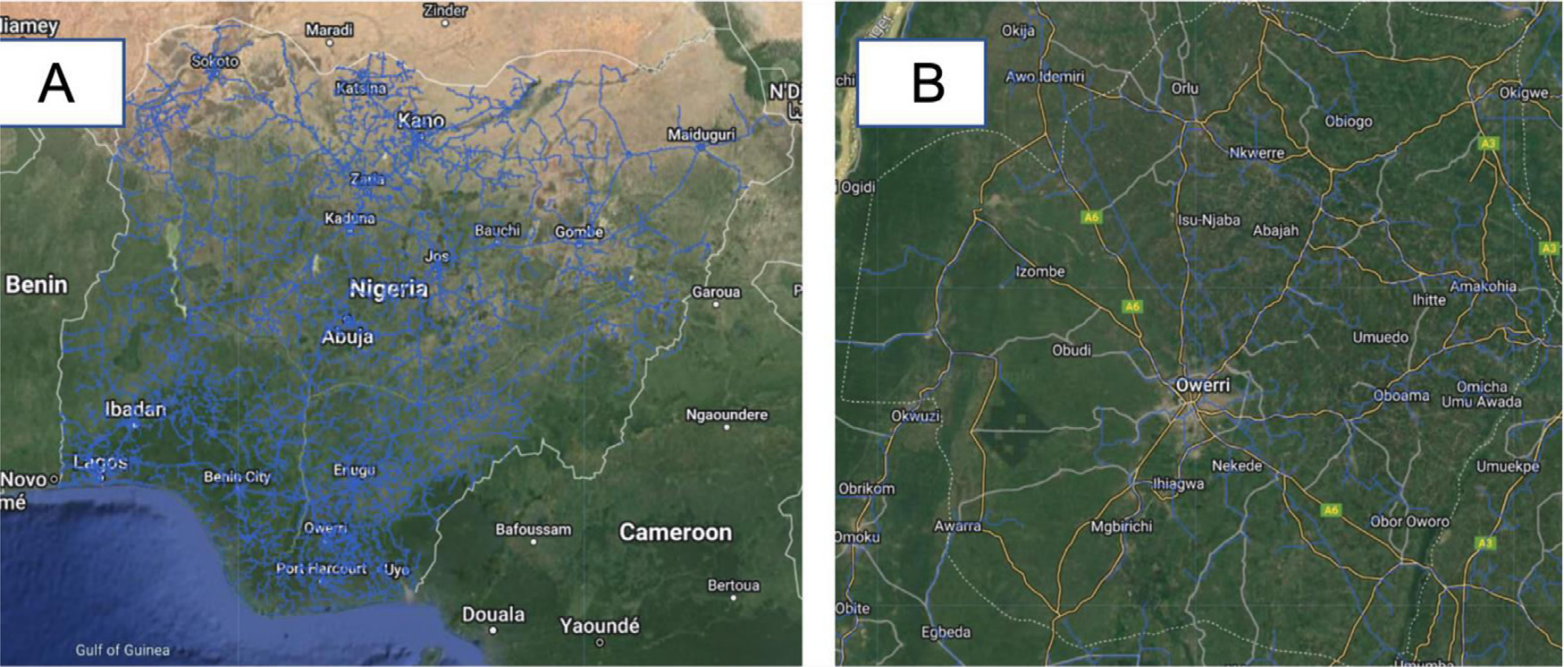
Predicted electricity network lines layer (A: Nigeria, B: Imo state (Terrain background with 80% opacity)). The blue color vectors represent the electrical transmission and distribution lines in Nigeria. The global electrical grid was sourced from Ref. [15] and clipped to the extent of Nigeria in QGIS (version 3.24 Tisler).

**Fig. 5:**
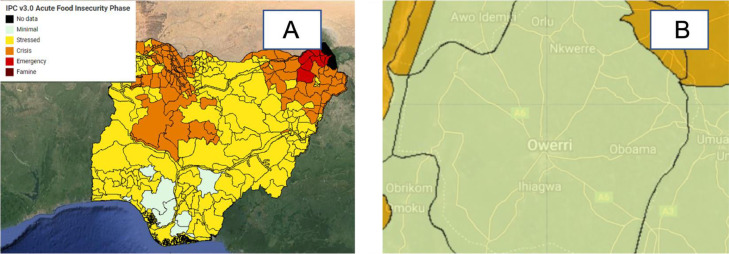
Acute food insecurity layer for the months in the range June – September for the year 2022 (A: Nigeria, B: Imo state (Terrain background with 80% opacity)). The Acute food insecurity shapefiles were sourced from Ref. [16].

**Fig. 6:**
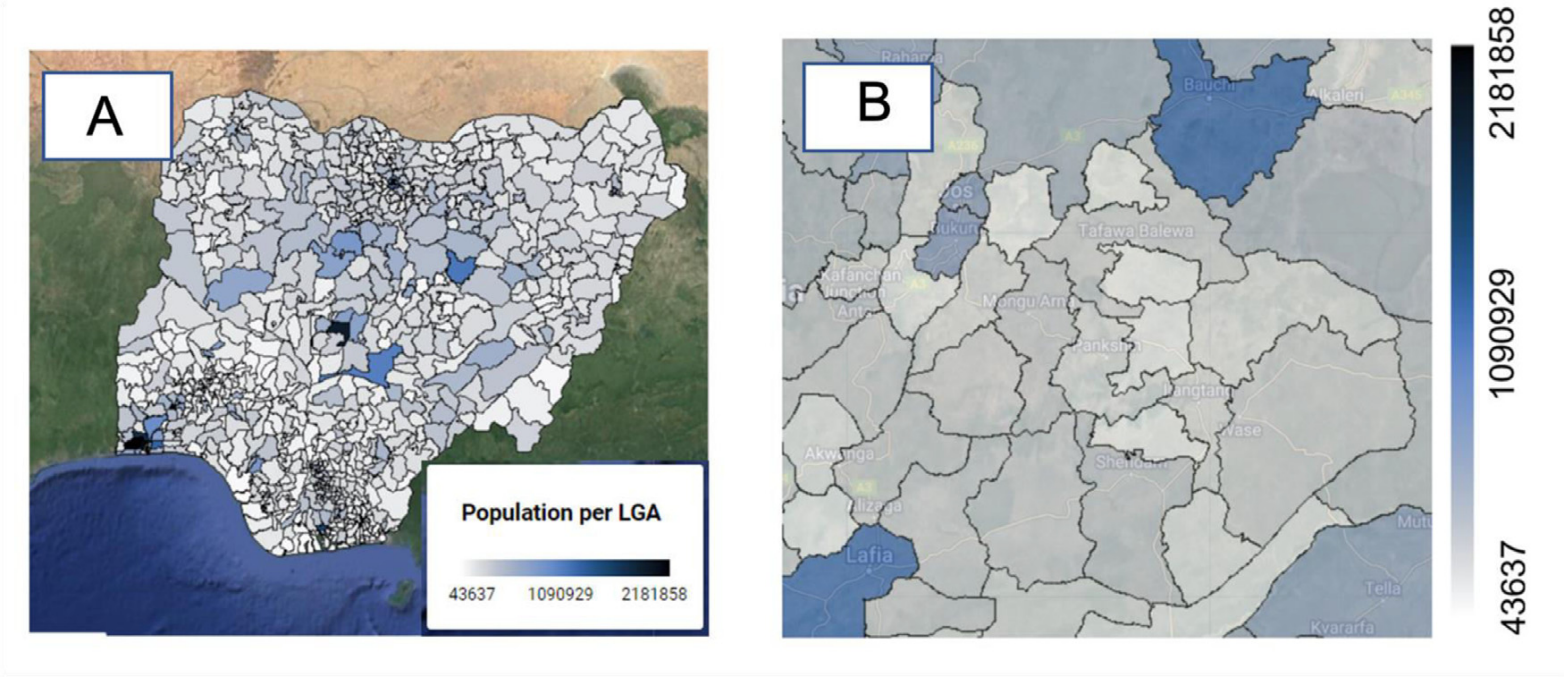
Mean LGA population layer (A: Nigeria, B: Plateau state (Terrain background with 80% opacity)). The population data per LGA in Nigeria was downloaded from Ref. [17].

#### Acute food insecurity phase layer

This layer describes the anticipated severity of acute food insecurity using the Integrated Food Security Phase Classification (IPC 3.0) developed by the Famine Early Warning Systems Network (FEWS NET) [16]. Datasets are available for download in Ref. [16].

#### Population per LGA layer

This layer displays the average population (in number of people) in each local government area (LGA) of Nigeria. The dataset was sourced from the official website of GRID3 Nigeria (Geo-referenced Infrastructure and Demographic Data for Development) [17].

#### Crop production layer

**Fig. 7:**
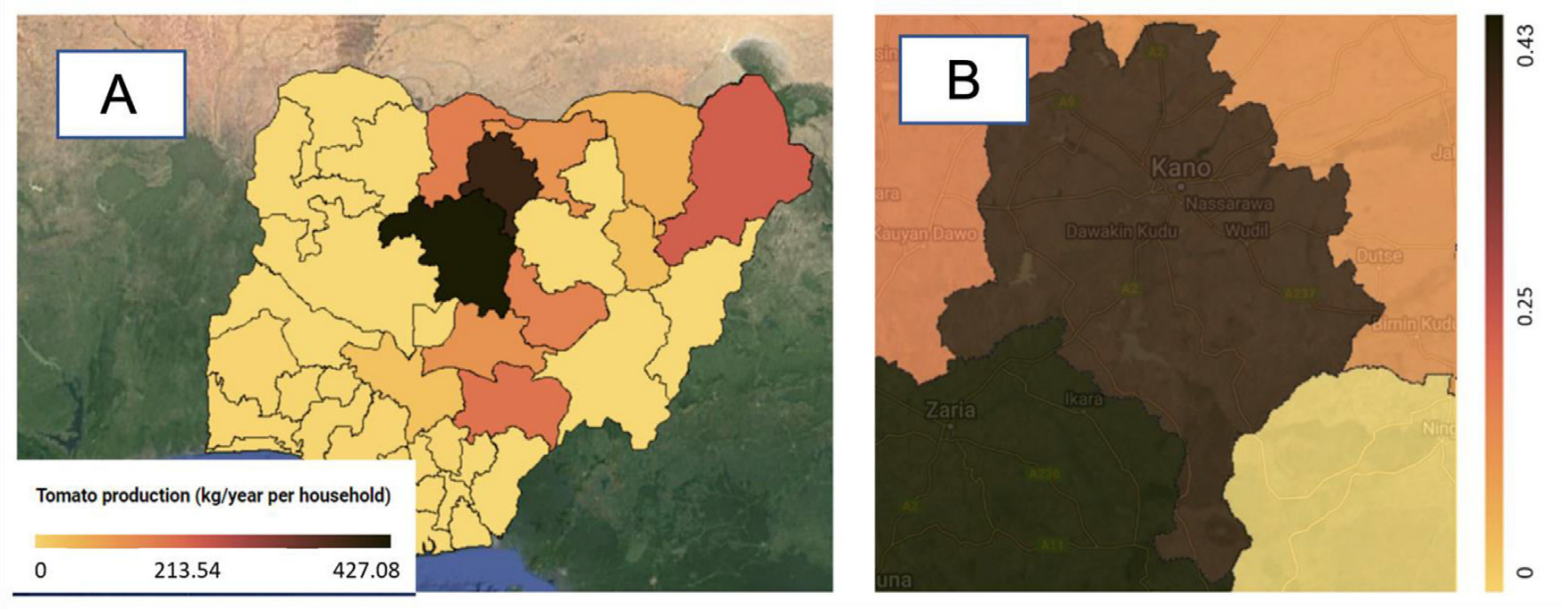
Crop production layer, an example tomato production (A: Nigeria, B: Kano state (Terrain background with 80% opacity)). The crop production tabular data with state statistics were sourced from Ref. [18].

The crop production layer displays production statistics for various fresh produce, including bananas, beans, cocoyam, cucumber, garden egg, melon, okro, onion, pepper, pineapple, plantain, potato, pumpkin, and tomato at a state-level spatial resolution. These data were sourced from the National Agricultural Sample Survey (NASS) of 2010. [18]. See Fig. 7 for an example of tomato production layer in KG per household for each state in Nigeria.

#### Agro-ecological zones layer

The Agro-ecological zones layer represents the grouping of Nigeria's agro-ecological zones into 13 classes. The data for this study was sourced from the Global Agro-Ecological Zoning version 4 (GAEZ v4) portal developed by the Food and Agricultural organisation (FAO) of the United Nations in collaboration with the International Institute for Applied System Analysis (IIASA) [19].

#### ESRI landcover layer

**Fig. 8:**
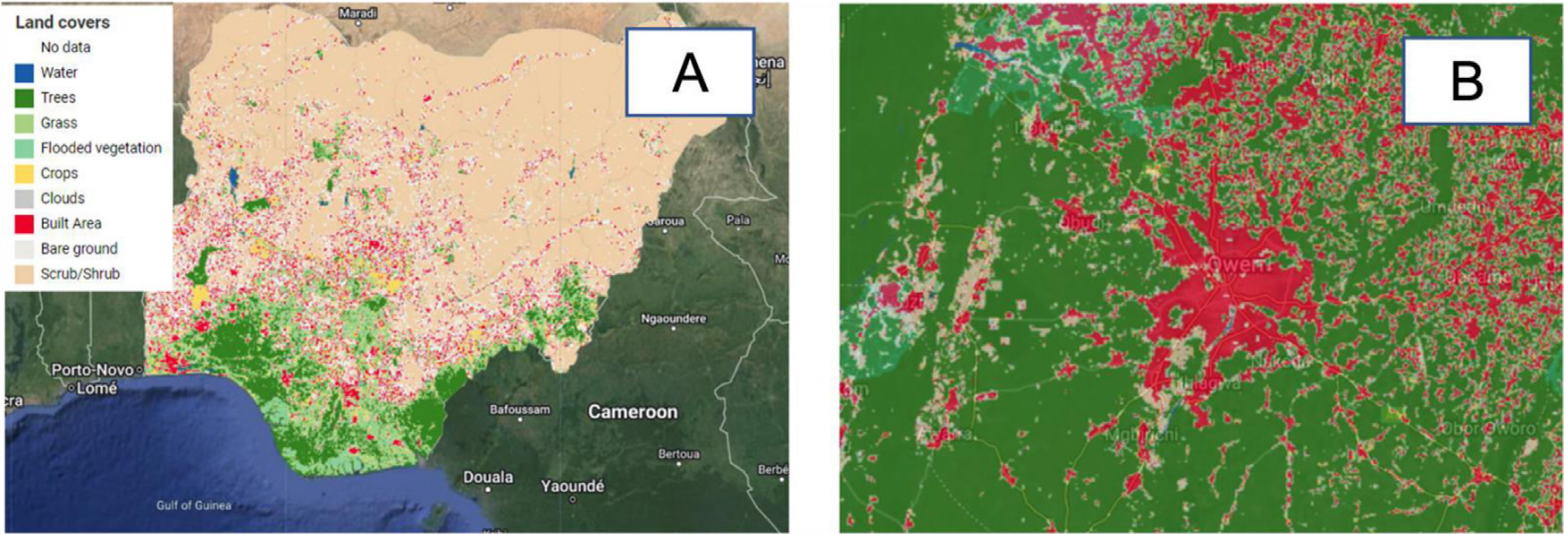
ESRI landcover layer (A: Nigeria, B: Imo state (Terrain background with 80% opacity)). The five landcover tiles used to generate this layer were sourced from Ref. [20].

The landcover layer displays the 10 classes of landcover in Nigeria as computed in Ref. [20] (Fig. 8). These landcover classes include, water, trees, grass, flooded vegetation, crops, clouds, built area, bare ground, and scrub-shrub. The layer was sourced from the Environmental Systems Research Institute (ESRI) geodatabase for global land cover [20].

#### Roads layer

**Fig. 9:**
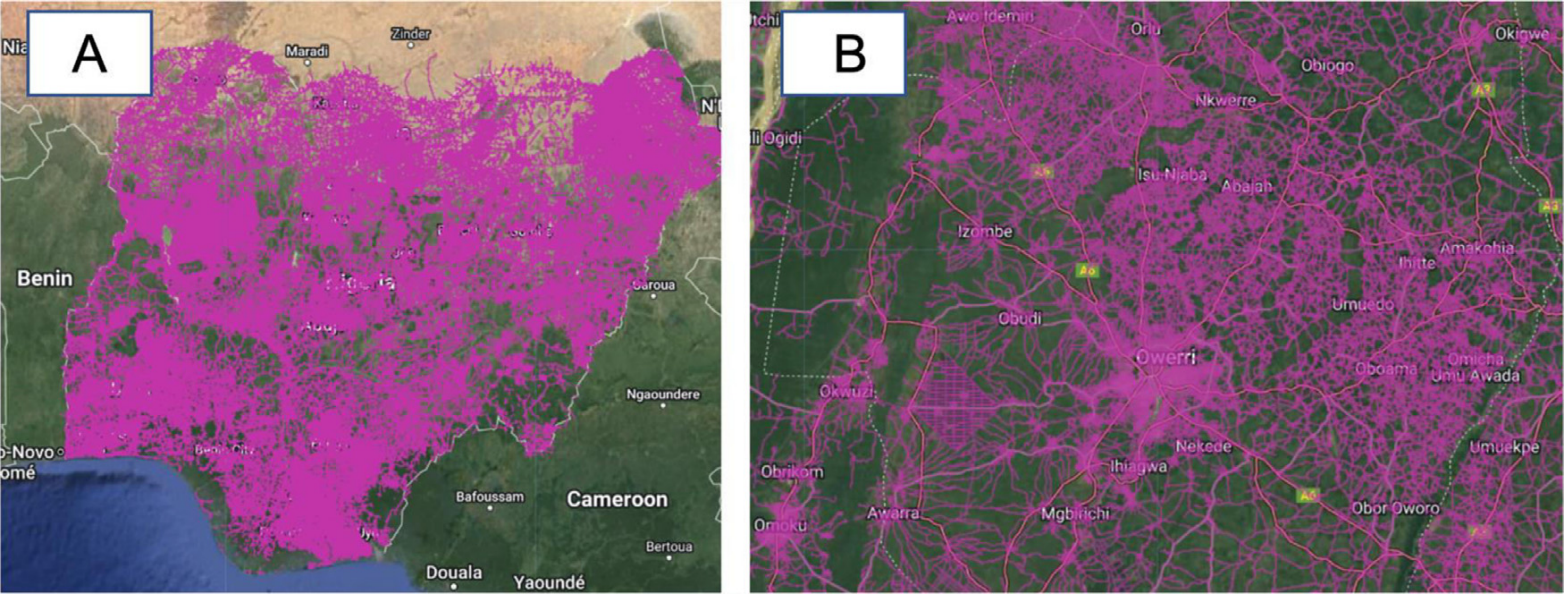
Roads layer, (A: Nigeria, B: Imo state (Terrain background with 80% opacity)). The dark pink color vectors represent the road networks in Nigeria. The Nigeria road network was downloaded from Ref. [21].

This layer represents the road network in Nigeria based on OpenStreetMap (Fig. 9). The dataset was downloaded from Ref. [21].

#### Market locations layer

**Fig. 10:**
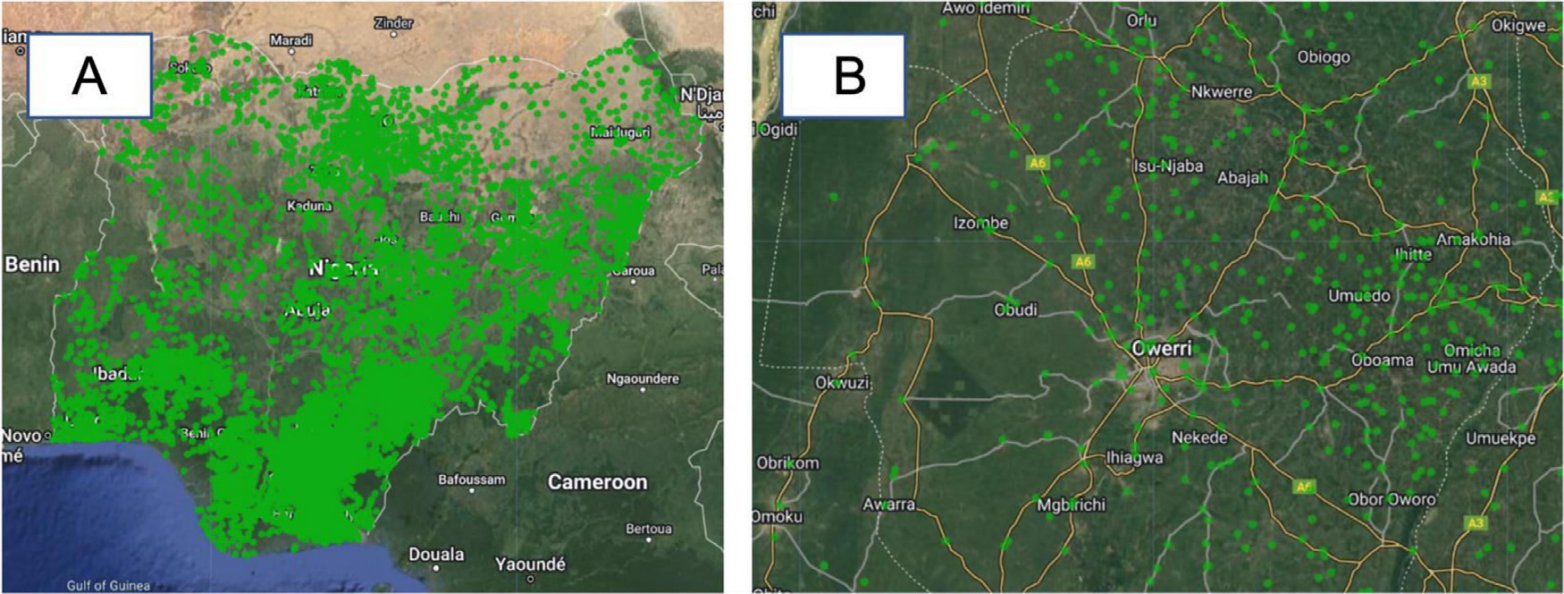
Market locations layer (A: Nigeria, B: Imo state (Terrain background with 80% opacity)). The green circular-shaped polygons represent the market locations in Nigeria. The market locations dataset was downloaded from Ref. [22].

This layer represents geolocated data corresponding to over 11,000 markets across Nigeria, comprising both formal and informal markets (Fig. 10). The dataset was sourced from GRID3 [22].

#### Market prices time series layer

**Fig. 11:**
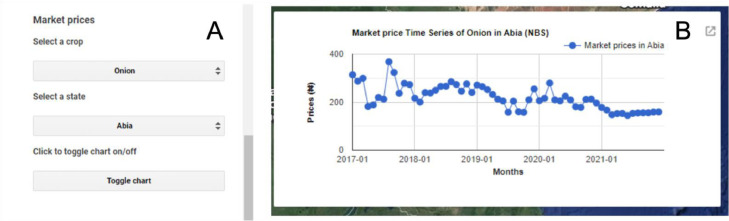
Market prices time series chart displaying the monthly market price time series for tomato in Abia state from January 2017 until December 2021 (A: Market prices panel, B: Market prices time series chart). The monthly market price tabular data were sourced from Ref. [23].

The aim of this layer is to visualize the monthly averaged market prices of certain fresh produce across Nigeria for the time period of 2017 till 2021 (Fig. 11). Commodities in this list include; onion, tomato, ripe plantain, unripe plantain, potato, and sweet potato. The data were downloaded from the E-library of the NBS [23].

#### Shelf life gain layer

This layer describes the gain in shelf life that can be achieved for selected commodities by keeping them at their optimal storage temperature instead of ambient temperature. Five key commodities produced in large quantities in Nigeria were selected for this layer, including tomato, bell pepper, cabbages, green beans, and carrots. The details of the calculations and models are discussed in Ref. [24].

#### Predicted cold room locations layer

**Fig. 12:**
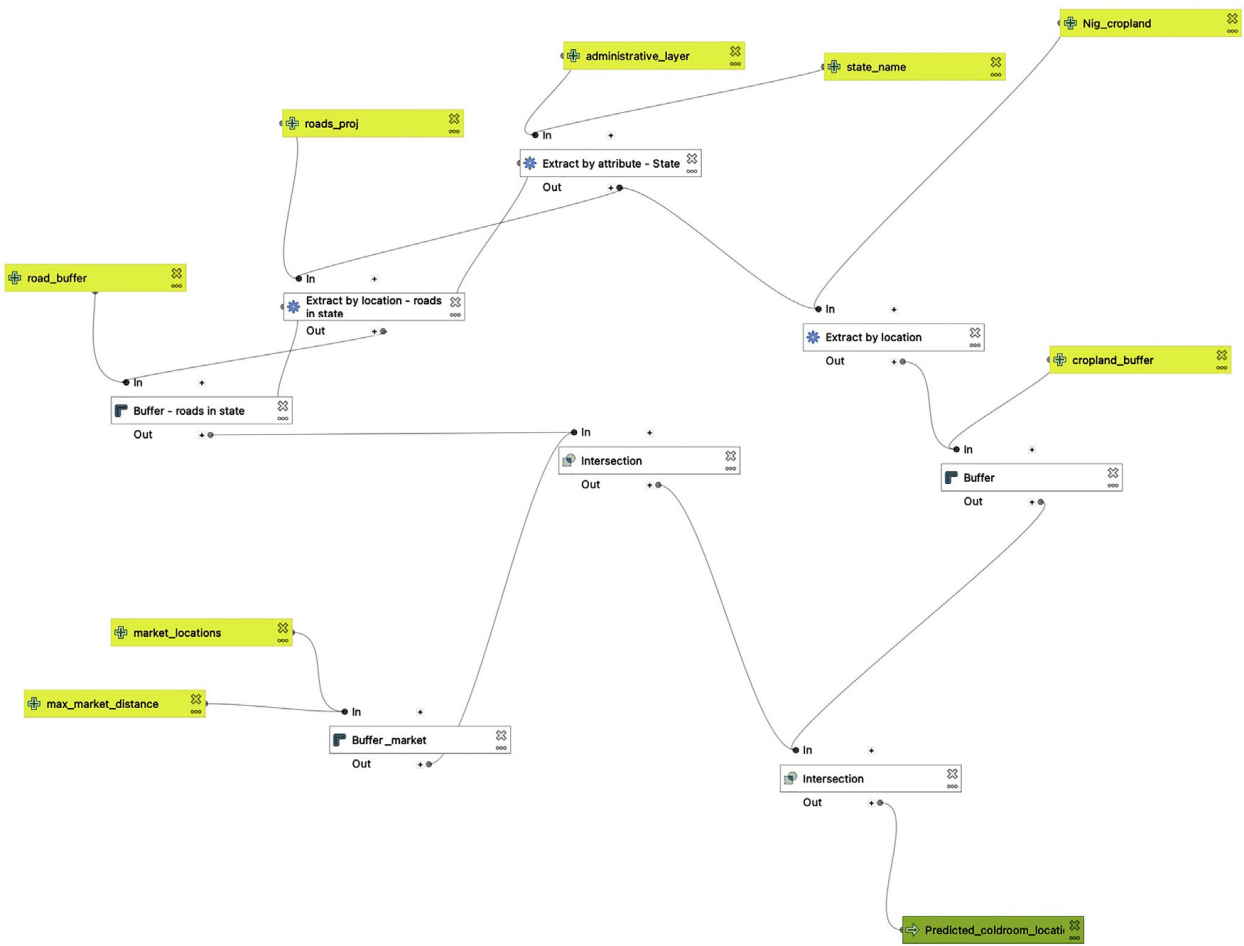
Graphical model used to generate the Predicted cold room locations for each state in QGIS (version 3.24 tisler). The yellow boxes represent the vector input layers, including the administrative layer showing state boundaries (Section 3.2), cropland (Section 3.3.16), roads (Section 3.3.12), and market locations (Section 3.3.13). The yellow boxes with “_buffer” attached to the titles represent the input buffer distance thresholds for the roads (Section 3.312), market locations (Section 3.3.13), and cropland (Section 3.3.16) layers. The white boxes represent the algorithms used to process these input layers. The green colored box represents the final Predicted cold room locations layer for each state (final output of the model).

**Fig. 13:**
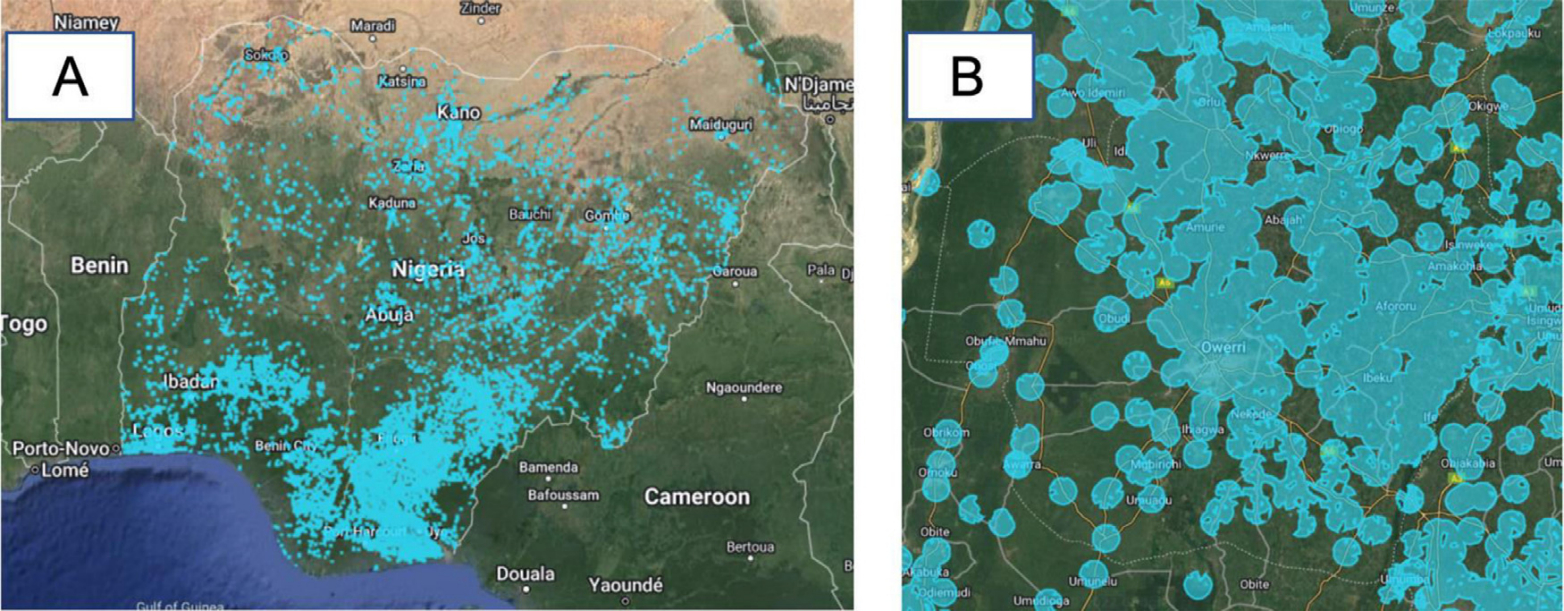
Potential cold room location layer (A: Nigeria, B: Imo state (Terrain background with 80% opacity)). The blue colored polygons represent the suitable areas in Nigeria for installing cold room facilities as computed by the Your VCCA team.

This layer on the web map represents suitable areas for siting cooling units in Nigeria based on important available open-source data (Fig. 13). Layers of importance were selected based on the need of a particular cooling service provider in Nigeria. This means that we used an algorithm that mimics the decision-making of a certain stakeholder to select areas that met certain pre-determined threshold criteria suitable for installing new cooling units. Some of these criteria required by a cooling service provider in Nigeria on which a threshold value is set, include; sun orientation, sunshine intensity, noise pollution, health hazard, land topography, market location and road accessibility. To calculate promising locations for installing new cold rooms, we used some layers discussed above in a model (See Fig. 12 ) developed on the Graphical modeler tool on QGIS (version 3.24 Tisler). The input layers for this model were the following:

• Roads (Section 3.3.12)
• Market locations (Section 3.3.13)
• ESRI Landcover (Section 3.3.11)

In this model, we made key assumptions derived from insights provided to us by a certain stakeholder to filter out areas in the map that did not meet said assumptions. These assumptions are as follows;

• We selected areas that are no more than 2 km from a market
• Areas that are at a distance of 500 m from a road (access route proximity)
• Areas that are no more than 2 km from cropland.

Note that this assumption may vary depending on the service provider and the geographical location of the country of interest.

The merged and clipped ESRI landcover raster discussed in Section 3.3.11 was used to generate a binary cropland raster where cropland pixels were represented with values of one and the rest classes with zeroes. This was done using the ‘raster calculator’ tool on QGIS (version 3.24 Tisler). This raster was then polygonized with the ‘polygonize’ tool on QGIS (version 3.24 Tisler) to generate a vector showing cropland features in Nigeria. Some of these features had invalid geometries so, the ‘Fix geometries’ tool on QGIS (version 3.24 Tisler) was used to correct these features. The resulting cropland vector is then used in the model to calculate the distance from cropland pixels. In this model, the state boundary for the selected state is extracted by state name from the state boundary shapefile [3] using the ‘Extract by attribute’’ algorithm from the processing toolbox in QGIS (version 3.24 Tisler), the roads in the selected state are extracted using the ‘Extract by location’ algorithm and the Roads layer (Section 3.3.12) as input. The same algorithm is used to extract the croplands in the selected state with the cropland vector as input. The markets and croplands in the selected state are then buffered by a distance of 2 km using the ‘Buffer’ tool in the processing toolbox in QGIS (version 3.24 Tisler), while a distance of 500 m buffers the roads. These buffered vectors are then intersected using the ‘Intersection’ algorithm to derive the final predicted cold room locations in the selected state. For the final step, the predictions for all 36 states, including the FCT, were merged into a single layer on QGIS (version 3.24 Tisler) and uploaded to Google Earth Engine. There are some limitations of this layer that are worth mentioning. The 2 km condition for markets is the major limiting factor for the output predicted locations of the model. Furthermore, note that this cropland vector is based on the Esri Landcover layer which is generated from machine learning models [22] and therefore is not completely accurate. Lastly, the roads layer (Section 3.3.12) sourced from Ref. [19] does not cover all roads in Nigeria and, therefore, might exclude some dirt roads.

This layer was created to suggest to cooling service providers the best areas for locating future cooling units. However, the Your VCCA team declines any responsibility for any errors or omissions in the information provided. For the next version of this application, we plan to introduce more interactivity by allowing users to compute layers using their own selected input layers and thresholds.

The terms of use for this dataset can be found here: Creative Commons Attribution 4.0 International

### Google Earth Engine web map

GEE is an open-source platform combining a multi-petabyte catalog of satellite imagery and geospatial datasets for planetary-scale analysis. The platform is available to the public *via* the link (GEE, https://earthengine.google.com/). The user interface (UI) template for organizing the code contained in the GEE script for this project was sourced from (the Google Earth YouTube channel, https://www.youtube.com/watch?v=saaecbImPmI. Following the instructions from the framework template specified in the video above, the code is divided into six sections; Model, Components, Composition, Styling, Behaviours, and Initialization sections, as follows:

1. Model: In this section, all the data displayed on the map and other relevant information are defined as objects in the code. This includes all Images, Image collections, Feature, and Feature collections. Image collections and Feature collections are stacks or sequences of related images and features respectively that are grouped together to allow operations to be carried out on the entire group, such as sorting and filtering. In GEE, raster data are uploaded as Images and Image collections, while vector data are uploaded as Features and Feature collections
2. Components: In this section, all the UI widgets are defined. Widgets are the components of the Graphical User Interface (map) that allow for user interactivity which include the panels, selectors, sliders, buttons, and charts. An example is the sliders on the map which allows the user adjust an integer value between 1 and 12 representing the 12 months in a year. The resulting effect is that the corresponding layer for the selected month is overlayed on the map
3. Composition: In this section, all the UI components defined in the components section above are arranged to give a complete UI. An example is the control panel located at the extreme left position of the web map. This scrollable panel contains all the widget groups for controlling how the layers are displayed. Here, all the other defined panels, including shelf-life gain, crop production, water access, water scarcity, market prices, climate panels, etc, are arranged to give a clean look and promote a great user experience (See Fig. 14).

**Fig. 14:**
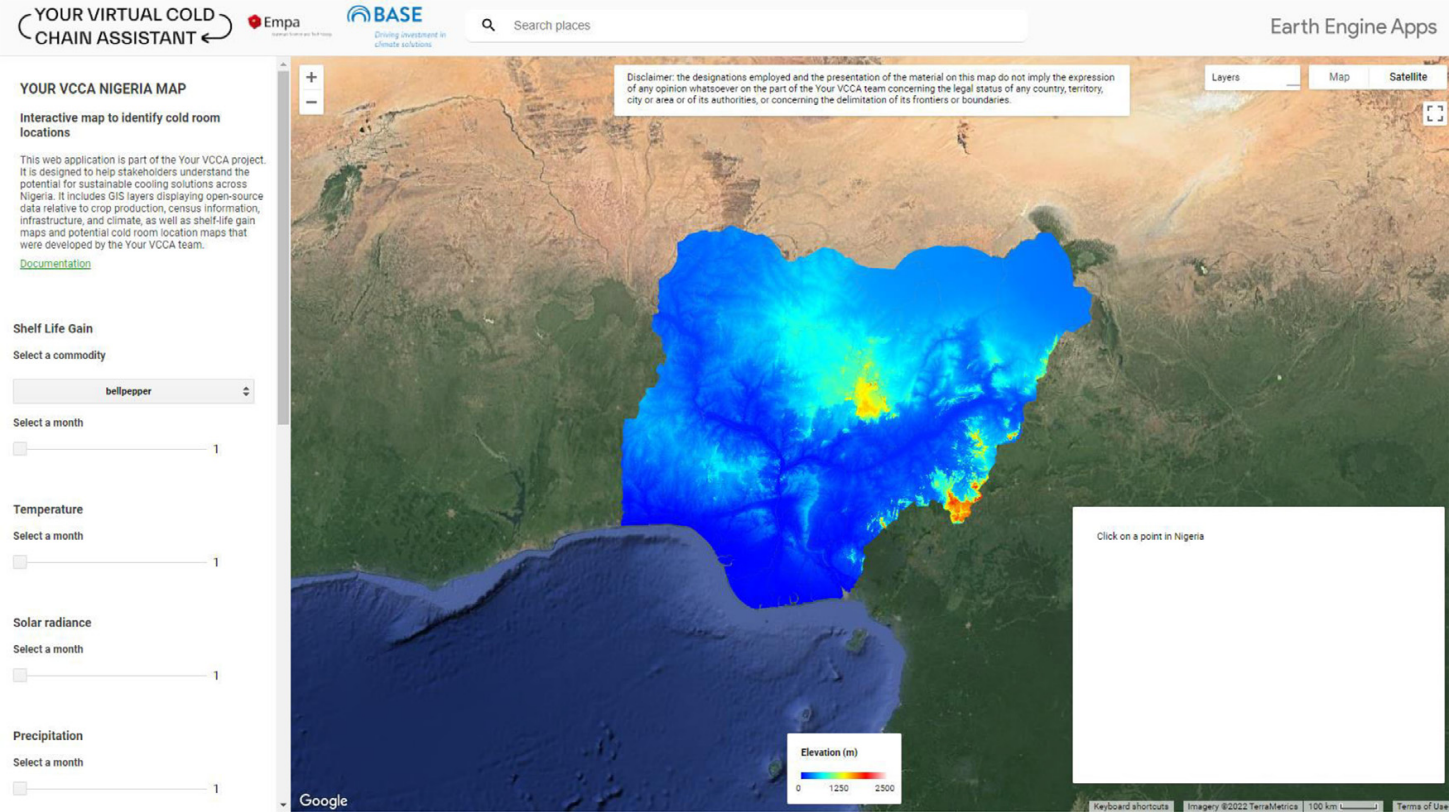
Google Earth Engine web map showing the elevation layer (Section 1.4.2).
4. Styling: The style properties of all widgets are defined in this section. Style properties describe simple mechanisms in the code for adding style, such as color, spacing, fonts, and position to the components of the application to make them look presentable to the user.
5. Behaviors: This section contains the majority of the programming functions that dictate how the app behaves when a user interacts with it. These functions are tied to the widgets using event listeners such as Onclick for buttons, and onChange for the selectors and sliders. Event listeners as their name implies are procedures in JavaScript that wait for specific events to occur in order to execute a function. An example of such events is a user clicking on a button
6. Initialization: Most applications include an initialization or entry point with defined properties. The initialization point is typically the first thing you see when you open an application. As for the web map, all the UI components and the Predicted cold room locations layer are displayed when a user loads the URL link on a browser. In the code of the web map, all except the topmost layer are kept invisible to improve the map loading speed.

The color palette for visualizing most of the layers was taken from the palette package and can be found here in the above link. The UI components for this web map are grouped into 4 major component groups, the control panel, the map, the value panel, and the legends.

## CRediT authorship contribution statement

**Divinefavour Odion:** Data curation, Investigation, Methodology, Formal analysis, Visualization, Resources, Writing – original draft, Writing – review & editing. **Kanaha Shoji:** Data curation, Investigation, Methodology, Formal analysis, Resources, Writing – review & editing. **Roberta Evangelista:** Data curation, Investigation, Formal analysis, Methodology, Resources, Writing – review & editing. **Joaquin Gajardo:** Data curation, Investigation, Methodology, Formal analysis, Resources, Writing – review & editing. **Thomas Motmans:** Conceptualization, Funding acquisition, Writing – review & editing. **Thijs Defraeye:** Conceptualization, Funding acquisition, Project administration, Writing – review & editing. **Daniel Onwude:** Project administration, Data curation, Investigation, Methodology, Funding acquisition, Writing – review & editing, Visualization, Resources, Writing – original draft.

## Declaration of Competing Interest

The authors declare that they have no known competing financial interests or personal relationships that could have appeared to influence the work reported in this paper.

## Data Availability

Data will be made available on request.
